# Pore forming polyalkylpyridinium salts from marine sponges versus synthetic lipofection systems: distinct tools for intracellular delivery of cDNA and siRNA

**DOI:** 10.1186/1472-6750-6-6

**Published:** 2006-01-16

**Authors:** Debra McLaggan, Noppadon Adjimatera, Kristina Sepčić, Marcel Jaspars, David J MacEwan, Ian S Blagbrough, Roderick H Scott

**Affiliations:** 1 School of Medical Sciences, College of Life Sciences and Medicine, University of Aberdeen, Institute of Medical Sciences, Foresterhill, Aberdeen AB25 2ZD, UK; 2 Department of Pharmacy and Pharmacology, University of Bath, Claverton Down, Bath BA2 7AY, UK; 3 Department of Biology, Biotechnical Faculty, University of Ljubljana, Veèna pot 111, 1000 Ljubljana, Slovenia; 4 Marine Natural Products Laboratory, Department of Chemistry, University of Aberdeen, Aberdeen AB24 3UE, UK

## Abstract

**Background:**

Haplosclerid marine sponges produce pore forming polyalkylpyridinium salts (poly-APS), which can be used to deliver macromolecules into cells. The aim of this study was to investigate the delivery of DNA, siRNA and lucifer yellow into cells mediated by poly-APS and its potential mechanisms as compared with other lipofection systems (lipofectamine and *N*^4^,*N*^9^-dioleoylspermine (LipoGen)). DNA condensation was evaluated and HEK 293 and HtTA HeLa cells were used to investigate pore formation and intracellular delivery of cDNA, siRNA and lucifer yellow.

**Results:**

Poly-APS and LipoGen were both found to be highly efficient DNA condensing agents. Fura-2 calcium imaging was used to measure calcium transients indicative of cell membrane pore forming activity. Calcium transients were evoked by poly-APS but not LipoGen and lipofectamine. The increases in intracellular calcium produced by poly-APS showed temperature sensitivity with greater responses being observed at 12°C compared to 21°C. Similarly, delivery of lucifer yellow into cells with poly-APS was enhanced at lower temperatures. Transfection with cDNA encoding for the expression enhanced green fluorescent protein was also evaluated at 12°C with poly-APS, lipofectamine and LipoGen. Intracellular delivery of siRNA was achieved with knockdown in beta-actin expression when lipofectamine and LipoGen were used as transfection reagents. However, intracellular delivery of siRNA was not achieved with poly-APS.

**Conclusion:**

Poly-APS mediated pore formation is critical to its activity as a transfection reagent, but lipofection systems utilise distinct mechanisms to enable delivery of DNA and siRNA into cells.

## Background

The technologies investigated in this study involved the use of polymeric alkylpyridinium sponge toxins, (polyalkylpyridinium salts; poly-APS) [1-3]. Poly-APS is a marine toxin preparation extracted from the sponge *Reniera sarai*, which can form reversible pores or lesions in cell membranes [1]. This toxin preparation primarily contains a cocktail of two polymeric 1,3-octylpyridinium salts of 5.5 and 19 kDa sizes [4]. At a high concentration (50 μg/ml), polyalkylpyridinium salts can cause irreversible depolarisation in membrane potential and a collapse in input resistance. However, at lower concentrations (0.5 μg/ml) poly-APS show reversible actions on the electrophysiological properties and Ca^2+ ^permeability in HEK 293 cells, dorsal root ganglion neurons and F-11 cells. The unique pore formation properties in addition to their poly-positive charged characteristics, could be useful in non-viral gene therapy (NVGT) applications. In a previous study [2] some us have shown that sponge toxins can be used to deliver cDNA into HEK293 cells and that this results in the expression of the protein encoded by these genes. Furthermore, the co-transfection of cDNA is stable and colonies of cells can be produced that contain poly-APS delivered cDNA and the proteins they encode for [2]. However, there has to date been no reported study on the interaction of this polycationic toxin with DNA and further information is required on the mechanism of transfection and the versatility of macromolecule (DNA or siRNA) delivery. This is particularly important given that our data suggest membrane poration is required for poly-APS-mediated transfection because low molecular weight pyridinium surfactant monomers and dimers can also be efficient non-toxic agents for gene delivery [8]. However, in contrast, pyridinium surfactant monomers are not efficient pore formers and appear to package DNA for delivery by fusion and/or endocytosis.

**Figure 1:**
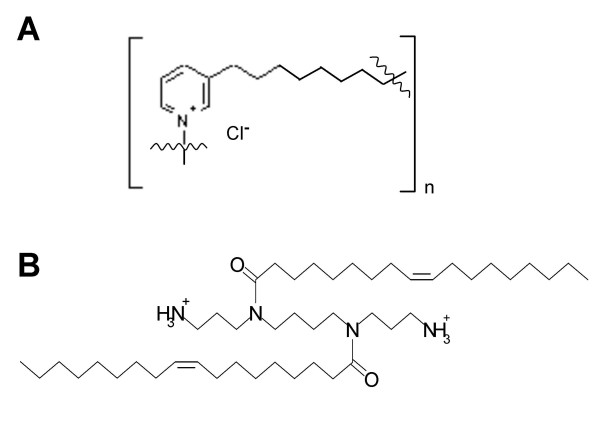
Structures of transfection reagents and other related molecules of interest. A, polymeric alkylpyridinium salt (poly-APS) a sponge toxin preparation composed of a mixture of two polymeric 1,3-octylpyridinium salts of 5.5 (n = 29 average) and 19 kDa (n = 99 average) sizes. B, *N*^4^,*N*^9^-dioleoylspermine (LipoGen).

The aims of our study were to explore the DNA condensation properties and temperature sensitivity of their gene delivery mechanism(s). In addition, the aims were extended to the delivery, using poly-APS, of small interfering RNA (siRNA) into cells and thus to the knockdown of gene expression. Comparisons are made with the activity of LipoGen, a synthetic lipospermine (see Figure 1) an efficient DNA delivery agent [5-7], and lipofectamine, a commercially available transfection reagent.

## Results

### DNA condensation study of poly-APS and LipoGen

The DNA condensation experiments were run with a plasmid encoding for enhanced green fluorescent protein (pEGFP) or luciferase (pGL3), and with calf thymus DNA (ctDNA). In DNA condensation experiments, ethidium bromide (EthBr), a DNA intercalating cationic dye was used as a fluorescent probe. This intercalation increases the fluorescence yield of EthBr (excitation 546 nm, emission 595 nm). Many compounds that bind to DNA, including DNA condensing agents used in NVGT, can displace EthBr from EthBr-DNA complexes. EthBr has been widely used to assess the DNA-polyamine complex formation efficiently [9,10]. In 2000, a modified reproducible EthBr displacement assay was reported by Geall and Blagbrough [9]. The excitation wavelength was optimised at 260 nm [11] where EthBr is indirectly excited by energy transfer from the DNA. The proposed protocol also allows the experiment to be run without pre-complexing DNA and its binding molecule, a "displacement assay" [9]. The DNA condensed particle formation has been measured at UV absorbance of 320 nm [12]. The double-helical DNA was bound by polyamines and formed nanoparticles, which scatter the light resulting in the UV absorbance increase above 300 nm. Light scattering (LS) is measured rather than UV absorption (no chromophore for this wavelength). A little precipitation of the DNA may be visible, but it does not increase the absorption above 300 nm. The DNA concentration used in this assay was a 10-fold excess compared to the EthBr assay given the low sensitivity of this experimental system and lack of fluorescence indicator.

**Figure 2:**
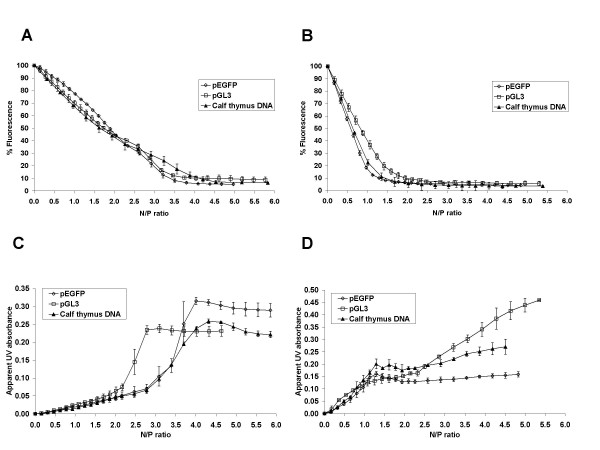
DNA condensation profile of poly APS and LipoGen using different DNA; pEGFP (4.7 kilobase pairs), pGL3 (5.3 kilobase pairs) and calf thymus DNA (13 kilobase pairs), using ethidium bromide displacement and light scattering assays. Ethidium bromide assay shows a decrease of fluorescence intensity (excitation at 260 nm, and emission at 600 nm) when poly-APS (A) or LipoGen (B) concentration was increased, expressed in a mole ratio of ammonium/DNA phosphate (N/P). Both poly-APS (4.1–4.7 μg/ml) and LipoGen (3.3–5.5 μg /ml) condense DNA efficiently with 10% residual fluorescence at N/P ratios 3.5 – 4.0 and (N/P) 1.5 – 2.5 respectively, (n = 3 for all experiments, error bars represent standard deviation). Light scattering confirmed that cationic vector-DNA complexes were formed, (C) for poly-APS, (D) for LipoGen, as the apparent absorbance at 320 nm increases significantly from the DNA only control solution.

Both poly-APS and LipoGen (Figure 1) were able to condense pEGFP, pGL3, and ctDNA efficiently with residual fluorescence at 10 % in ethidium bromide (EthBr) assay (Figure 2A,B). The increase of UV absorption was also confirmed by light scattering experiments, indicating DNA nanoparticle formation (Figure 2C,D). Optimal N/P ratios for DNA condensation for poly-APS and LipoGen were at 3.50–4.00 (4.1–4.7 μg/ml) and 1.50–2.50 (3.3–5.5 μg /ml). All three DNA samples used in our study were condensed in the same manner by each compound. Interestingly, fluorescence intensity from LipoGen-mediated DNA condensation experiment decreased much more sharply than with poly-APS. This superior DNA condensation effect could be associated with the positive charge distribution/number and lipophilicity of LipoGen over poly-APS.

### Poration of cell membranes by poly-APS

**Figure 3:**
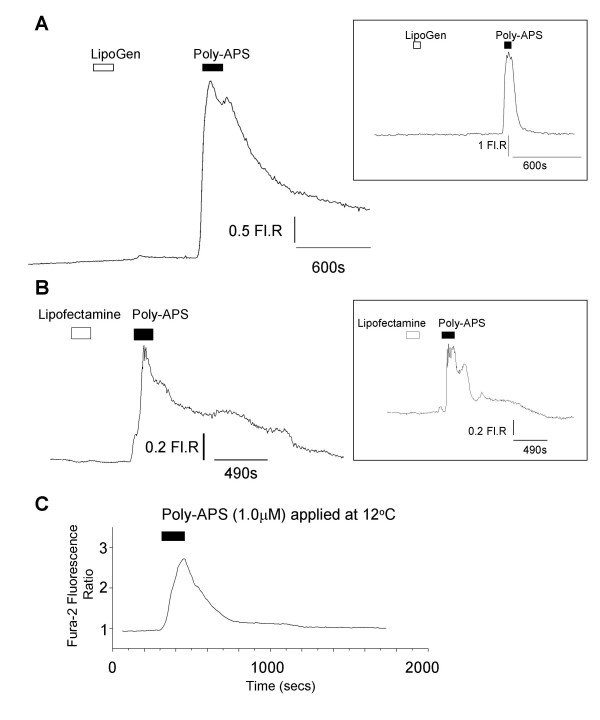
Records of fura-2 Ca^2+ ^imaging. A, average (mean; n = 30 HEK 293 cells) trace showing that LipoGen (1 μg/ml) failed to cause a rise in intracellular Ca^2+ ^but that polymeric alkylpyridinium salt (Poly-APS; 1 μg/ml) increased intracellular Ca^2+ ^measured as a change in fluorescence ratio (Fl.R). The rise in intracellular Ca^2+ ^was indicative of pore formation. Inset record shows a trace of changes in intracellular Ca^2+ ^from a single cell. B, average (mean; n = 19 HEK 293 cells) trace showing that lipofectamine (1 μg/ml) failed to cause a rise in intracellular Ca^2+ ^but that polymeric alkylpyridinium salt (Poly-APS; 1 μg/ml) increased intracellular Ca^2+^, indicative of pore formation. Inset record shows a trace of changes in intracellular Ca^2+ ^from a single cell. C, single cell trace of a Ca^2+ ^transient evoked at 12°C by poly-APS.

The measurement of increases in intracellular Ca^2+ ^with fura-2 can be used as an index of Ca^2+ ^entry through channels resulting from the interactions of pore forming molecules with cell membranes [1-3]. Ratiometric intracellular Ca^2+ ^imaging was conducted to compare pore formation or permeabilisation of cell membranes by poly-APS, LipoGen and lipofectamine. Poly-APS (1 μg/ml) evoked transient increases in intracellular Ca^2+ ^in HEK 293 cells at room temperature (21°C). In contrast LipoGen (1 μg/ml) and lipofectamine (1 μg/ml) both failed to evoke any changes in intracellular Ca^2+ ^indicating that acute application of these compounds at this concentration did not result in pore formation in cell membranes and an increase in Ca^2+ ^permeability (Figure 3A,B). Poly-APS also efficiently evoked Ca^2+ ^transients at 12°C (Figure 3C).

**Figure 4:**
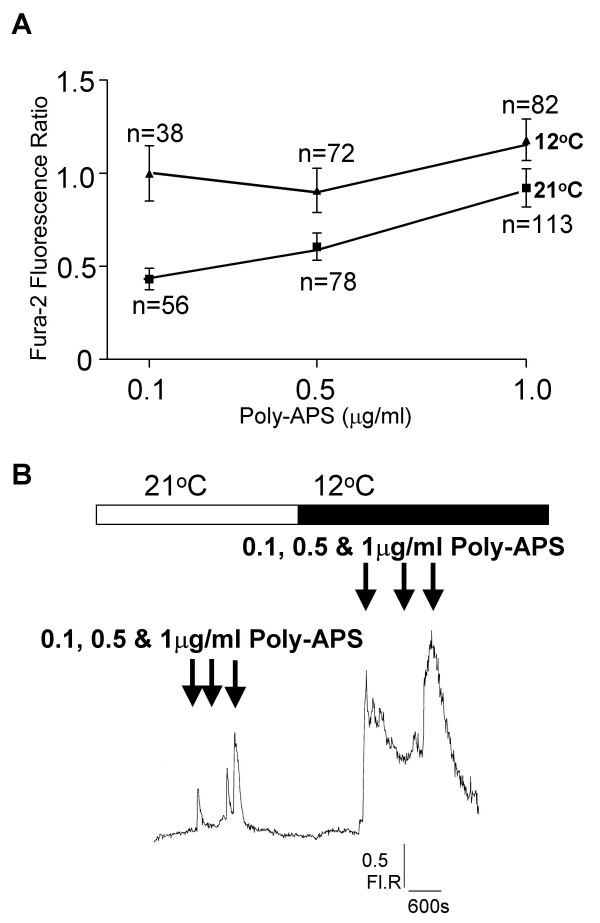
Temperature sensitivity of poly-APS actions. A, dose/response relationship for poly-APS-evoked Ca^2+ ^transients at room temperature (21°C, filled squares) and at 12°C (filled triangles). At each concentration of poly-APS, larger responses were obtained at the lower temperature. The different n values reflect variable sensitivities of HEK 293 cells to poly-APS with cells being more sensitive at lower temperatures. B, an example record showing larger increases in intracellular Ca^2+ ^(Fl.R Fluorescence Ratio units) in response to poly-APS at 12°C compared with 21°C.

Dose/response relationships for poly-APS-evoked intracellular Ca^2+ ^transients were generated at 12°C and at room temperature (21°C). At 21°C a clear dose-dependent relationship was obtained with 1.0 μg/ml poly-APS giving significantly larger responses than 0.1 μg/ml poly-APS (P < 0.005, n = 113 & 56). In contrast at 12°C no clear dose-dependent relationship was observed with the mean responses to 1.0 and 0.1 μg/ml poly-APS not being significantly different. This can be explained by the significantly larger Ca^2+ ^transients evoked by 0.1 μg/ml poly-APS at 12°C. At 12°C the peak Ca^2+ ^transient had a mean amplitude of 1.0 ± 0.15 fluorescent ratio units (n = 38), at 21°C the same concentration of poly-APS evoked a significantly smaller response of 0.43 ± 0.06 fluorescent ratio units (n = 56; P < 0.001; Figure 4A,B). This effect of temperature became less apparent at higher concentrations of poly-APS. At 1.0 μg/ml poly-APS there was no significant difference between responses obtained at 12°C and 21°C (Figure 4A). Therefore there was an apparent 10-fold shift in sensitivity to poly-APS with a 9°C drop in temperature. The increase in sensitivity of HEK 293 cells to poly-APS at low temperature was also apparent because a few cells failed to respond at all to poly-APS applied at 21°C but subsequently responded well to poly-APS when the temperature was reduced to 12°C. The majority of experiments were conducted by applying poly-APS at room temperature and then reducing the temperature to 12°C and reapplying cooled poly-APS. However, temperature sensitivity was still equally apparent when the experiments were conducted the other way around by firstly applying poly-APS at 12°C and then raising the temperature to 21°C and reapplying poly-APS. The changes in temperature sensitivity to poly-APS were readily reversible within 5 to 10 min of a temperature increase or decrease.

### Intracellular delivery of lucifer yellow

**Figure 5:**
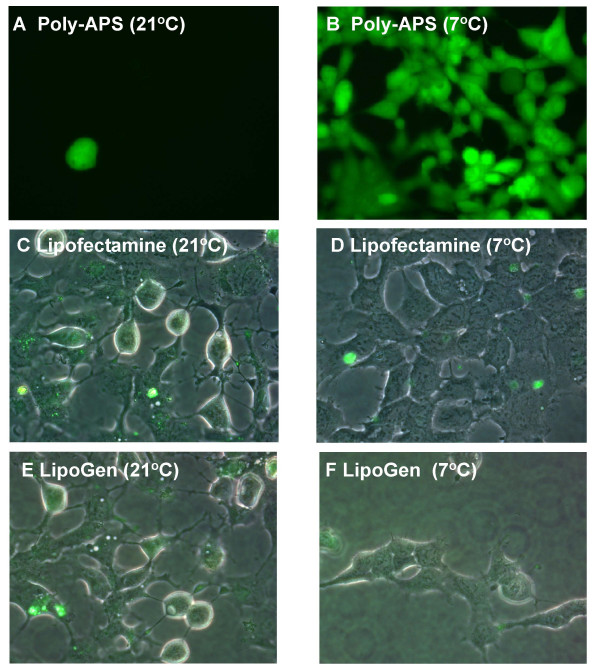
Temperature effects were also seen when poly-APS was used for intracellular delivery of lucifer yellow. In the absence of poly-APS no intracellular uptake of lucifer yellow was observed over a 3 h incubation period. After incubation with poly-APS and lucifer yellow (1 mM) for 3 h at 21°C a few individual cells were loaded (A), but after incubation at 7°C almost all cells are filled with lucifer yellow (B). Lipofectamine and LipoGen did not deliver lucifer yellow efficiently. Merged transmission and fluorescence images show that a very modest amount of fluorescence was observed after incubation with lipofectamine and lucifer yellow (1 mM) for 3 h at 21°C (C) and no cells were loaded at 7°C (D). Similarly, with LipoGen, little fluorescence was seen after incubation at 21°C (E) and no cells were loaded at 7°C (F).

Experiments into pore formation were also conducted using lucifer yellow as a probe. Although lucifer yellow has a low molecular weight (457.25 Da) it is a convenient dye to assess changes in membrane integrity. In the absence of poly-APS, no lucifer yellow was found intracellularly, this was irrespective of the incubation periods of 1, 2 and 3 h and temperature, 7°C, 21°C and 37°C. Similarly, no intracellular delivery of lucifer yellow was achieved using poly-APS at an incubation temperature of 37°C. However, low levels of intracellular delivery of lucifer yellow, was seen with poly-APS at an incubation temperature of 21°C. We estimate that at 21°C intracellular loading with lucifer yellow was seen in about 5 % of HEK 293 cells, but that this loading into individual cells was intense (Figure 5A). In contrast, when incubations were carried out at 7°C poly-APS achieved efficient loading of lucifer yellow into at least 90 % of cells (Figure 5B). Furthermore, the loaded lucifer yellow remained in these cells after returning the cells to culture medium and incubating them in standard culture media at 37°C for 24 h. In contrast little (21°C) or no (7°C) lucifer yellow uptake was observed when HEK 293 cells were incubated with either lipofectamine (4 μg/ml; Figure 5C,D) or LipoGen (4 μg/ml; Figure 5E,F).

### pEGFP delivery by poly-APS and lipopolyamines

**Figure 6:**
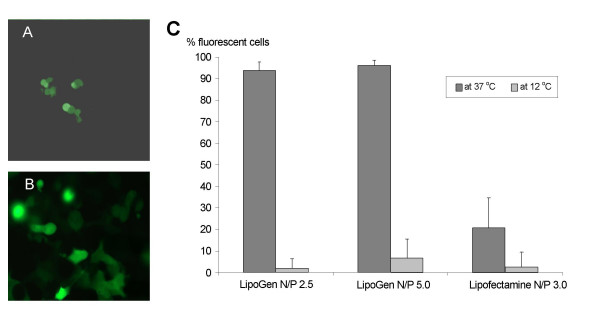
Effect of temperature on gene delivery. A, confocal image showing expression of enhanced green fluorescent protein in HEK 293 cells after transfection at 12°C with poly-APS (0.5 μg/ml; 2.5 μg pEGFP). B, fluorescence microscope image showing expression of enhanced green fluorescent protein in HEK 293 cells after transfection at 12°C with lipofectamine (4 μg/200 μl; 1 μg pEGFP). After incubation at 12°C for cDNA delivery, cells were returned to standard culture media and conditions and incubated at 37°C for 24 h before being examined. C, Histogram showing the temperature dependence of gene delivery to HtTA HeLa cells. pEGFP DNA complexed with LipoGen (N/P ratio 2.5 and 5.0) and Lipofectamine (N/P ratio 3.0) were added into each well of HtTA HeLa cells (cervix carcinoma, 50% confluent) in serum-free media. Transfection experiments were performed at 37°C or 12°C, and then DNA complexes were removed after 4 h exposure. The number of fluorescent cells was determined by FACS cytometry after 44 h post-transfection at 37°C, to determine the efficiency of pEGFP delivery systems. Significant decrease of transfection efficiency at 12°C was found in all lipofection systems used, compared with standard transfection conditions at 37°C. (n = 3 for all transfections, three replicates each, error bars represent standard deviation).

Given that pore formation in cell membranes could be achieved at 12°C with poly-APS, we examined whether lipofectamine, LipoGen and poly-APS would still function as transfection reagents at lower temperatures. For these experiments different incubation protocols were used for lipofection systems and for poly-APS. A standard protocol forming complexes between lipofectamine and DNA and LipoGen and DNA was used but for poly-APS to work poly-APS was applied separately to cells to allow pore formation before adding DNA [2]. Incubation protocols with HEK 293 cells, lipofectamine and poly-APS at 12°C still resulted in delivery of cDNA encoding for the expression of enhanced green fluorescent protein and transfection with both reagents (Figure 6A,B). Lipofectamine was less efficient as a transfection reagent at 12°C with about 30–50 % of HEK 293 cells transfected compared with 75–80 % transfection at 37°C. With poly-APS the transfection efficiency was 20–30 % at 12°C and therefore similar to that achieved at 37°C [2]. Lipofection systems were also assessed at 12°C and 37°C using the same transfection condition and HtTA HeLa cells. There was a significant decrease of gene expression at 12°C (Figure 6C), compared to transfection and gene expression at 37°C normally used for optimal cell growth. This data suggests that LipoGen and lipofectamine-mediated transfection requires endocytosis, and probably does not work to any great degree through the pore formation mechanism.

### Intracellular delivery and function of siRNA

Given the abilities of poly-APS, lipofectamine and LipoGen to act as transfection reagents for cDNA we were interested to determine whether they would deliver siRNA into HEK 293 cells. For these experiments initial incubation protocols were carried out at 37°C because at this temperature transfection with cDNA has been achieved with all three reagents used. The intracellular delivery of siRNA was detected firstly by visualisation of intracellular siRNA labelled with fluorescein and secondly by evaluating knockdown of specific protein expression. Experiments using lipofectamine showed that in the presence of serum substantial knockdown in protein expression was observed after 48 h. In contrast when incubations were carried out in serum-free conditions substantial knockdown could be identified after 24 h but knockdown did not increase further after 48 h. Therefore in all other experiments serum-free incubation conditions were used and analysis of protein knockdown was carried out after 24 h.

**Figure 7:**
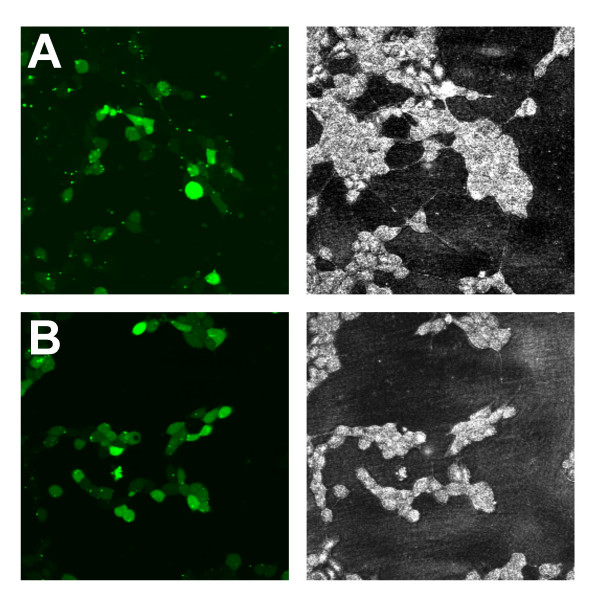
Delivery of siRNA with lipofectamine and LipoGen into HEK 293 cells. Intracellular delivery of siRNA fluorescein conjugate using A, lipofectamine and B, LipoGen as transfection reagents was visualised as confocal transmission images after a 4.5 h incubation period. No siRNA-positive cells were observed in the absence of a transfection reagent or after incubation with poly-APS. The left-hand images are fluorescent images and the right-hand images are corresponding transmission images.

**Figure 8:**
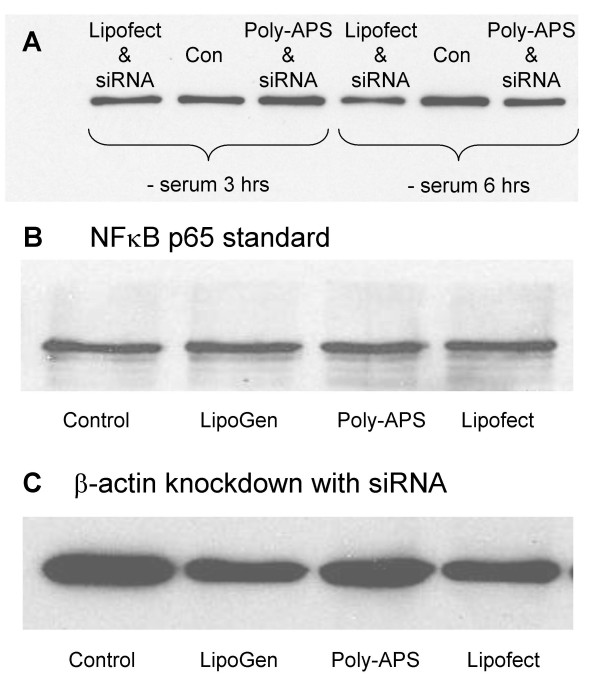
Delivery of β-actin siRNA using lipofectamine knocked down β-actin. A, Western blots of β-actin expression in HEK 293 cells and knockdown by delivery of β-actin siRNA using lipofectamine (Lipofect), but not poly-APS, in the absence of serum in the incubation media for 3 h and 6 h. The presence of 10 % serum has been shown to attenuate poly-APS-mediated pore formation, so experiments were conducted in serum-free conditions. B, shows a standard loading record for NFKB for the Western blots of siRNA knockdown of β-actin by LipoGen, poly-APS and lipofectamine (Lipofect) illustrated in part C of this figure. C, Western blots for siRNA knockdown of β-actin by LipoGen, lipofectamine (Lipofect) and poly-APS.

**Figure 9:**
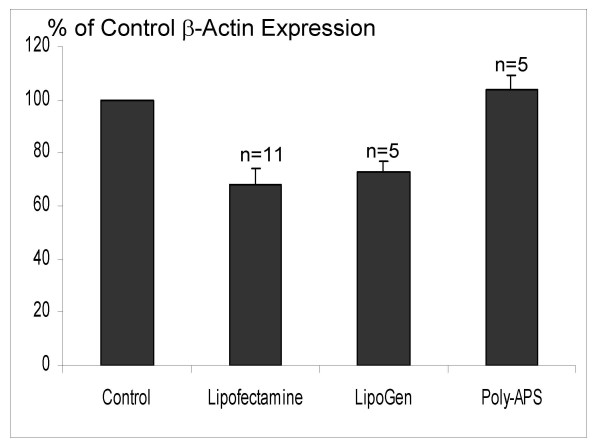
Histogram showing normalised data for siRNA knockdown of β-actin and the effect of different transfection reagents. All data are represented as mean percentage expression relative to the control. Data analyses conducted after 24 or 48 h are included.

Figures 7A&B show intracellular fluorescence produced by the fluorescein-labelled siRNA delivered into HEK 293 cells using lipofectamine or LipoGen. No intracellular fluorescence was observed under control conditions, in the absence of any transfection reagent (cells incubated with siRNA alone) or when 1.0 μg/ml poly-APS was used to porate the cell membrane. In the presence of poly-APS intracellular fluorescence of fluorescein-labeled siRNA was not seen even if the siRNA concentration was increased 4-fold. Additional experiments were conducted at a lower temperature to increase poly-APS-mediated pore formation. When incubations were carried out with poly-APS and siRNA conjugated with fluorescein at 12°C for 5 h no intracellular delivery of the siRNA was detected (n = 4). The presence or absence of 10 % serum did not influence either the activity of lipofectamine or the inactivity of poly-APS (Figure 8A). The delivery of siRNA specific for β-actin, using lipofectamine or LipoGen had functional consequences, knocking down protein expression by 30 % (max knockdown 34 & 65 % respectively) compared to β-actin expression in control cells (Figure 8B,C). The levels of β-actin seen in cells exposed to poly-APS and siRNA specific for β-actin were comparable with those measured for the controls but significantly (*P *<*0.005*) higher than the levels seen after lipofectamine or LipoGen mediated delivery of siRNA (Figure 9).

## Discussion

Poly-APS is a highly efficient DNA condensing agent with an additional property of being able to reversibly form pores in membranes, which are highly desirable characteristics for any non-viral vectors. In addition to cell entry facilitation, this membrane perturbation could also help in endosomal escape process, which is one of key barriers in gene delivery. The structure modification of poly-APS i.e. lipid conjugation, alkyl chain length control and degree of polymerisation, may further improve its DNA condensing, pore formation, transfection efficiency, and toxicity profiles.

Other pyridinium compounds have proved useful transfection reagents, these include the SAINT (synthetic amphiphile INTeraction) series [8], which have high transfection efficiency when used in a liposomal formulation with 1,2-dioleoyl phosphatidylethanolamine (DOPE). The SAINT series are conjugations of two fatty acid chains (tail group) with pyridinium head groups. From structure-activity relationship studies, it was found that unsaturated fatty chains enhanced the transfection efficiency. Head group modification by introducing another pyridinium group linked with an alkyl spacer, also significantly improved the transfection efficiency. Interestingly, C4 spacer (in SAINT-3) linking two pyridinium heads shows the highest efficiency in DNA delivery, compared to C3 and C5 spacers [8]. Lipophilicity and alkyl chain length modifications of poly-APS, leading to optimal positive charge regiochemical distribution, (distance between pyridinium head-groups in the poly-APS molecules) may in the future improve pore formation and transfection properties of modified poly-APS.

Previously, we have shown that a sponge toxin preparation, poly-APS, can produce pores in cell membranes and perform as a transfection reagent. This is unusual given that poly-APS is a natural product preparation and it raises the possibility that in nature as well as acting in chemical defence, polymeric alkypyridinium compounds may also provide a novel mechanism of natural horizontal genetic material transfer between marine microorganisms. The mechanism by which poly-APS delivers macromolecules into cells and achieves transfection is unknown. However, reversible pore formation by poly-APS and movement of macromolecules down concentration or electrochemical gradients into cells through these structures may provide a novel mechanism for the intracellular delivery of normally impermeant molecules. An alternative mechanism might involve the binding of poly-APS to cDNA, this complex binding to cell membranes and then internalisation by endocytosis. The processes of endocytosis are temperature dependent as temperature affects both rate of ligand binding and mobility of ligand-receptor complexes in membranes. Endocytotic processes are usually blocked at temperatures below 12°C. For example lucifer yellow uptake into platelets by fluid phase endocytosis occurs above 15°C [13]. Although platelets clearly load with lucifer yellow at 37°C no such uptake was seen at this temperature in HEK 293 cells. However, dramatic stable intracellular loading of lucifer yellow into HEK 293 cells was facilitated by poly-APS during low temperature incubations. This event was not supported by lipofectamine or LipoGen and must therefore be due to some processes other than fluid phase endocytosis. Experiments were conducted to determine whether poly-APS would permeabilise cells, as reflected by calcium influx, support transfection of cDNA encoding for the expression of enhanced green fluorescent protein and delivery of lucifer yellow into cells at 7–12°C. At low temperatures, poly-APS functions well, both as a pore former and as a transfection reagent for delivery of cDNA. This indicates that endocytosis is unlikely to be the mechanism by which poly-APS delivers cDNA and lucifer yellow into cells. Additionally, the protocol used for cDNA delivery also suggests that endocytosis is not involved because there is a requirement to incubate cells in poly-APS prior to exposure with cDNA. Furthermore, the temperature data adds to the evidence (pre-mixing poly-APS with cDNA actually prevents transfection), that pore formation by poly-APS is critical for macromolecule delivery. Although lipofectamine and LipoGen could be used as a transfection reagent at 12°C they were much less efficient at low temperatures compared to high temperatures. A further difference was the failure of lipofectamine and LipoGen to cause an increase in intracellular Ca^2+^, therefore these reagents show very different biological activity compared to poly-APS.

The polymeric structure of poly-APS appears critical to at least some of its biological activities. We previously found that two monomeric compounds, cetylpyridinium chloride and cetyltrimethylammonium bromide, did not reversibly permeate cell membranes and did not support transfection [14]. A recent investigation on poly-APS and synthetic linear alkylpyridinium monomers, dimers and tetramers showed that biological activities such as antibacterial, hemolytic activities, anti-acetylcholinesterase activities and inhibition of protein phosphatase 2A are greatly and differentially influenced by increasing numbers of positive charges and pyridinium rings. The smaller synthetic compounds showed good anti-enzymatic and antibacterial activity. In contrast poly-APS showed much greater hemolytic activity compared to the smaller synthetic compounds [15].

Evidence suggests that a reduction in temperature enhances pore formation by poly-APS both at the lowest concentration of poly-APS tested (0.1 μg/ml) and in cells that were less sensitive and failed to respond to poly-APS at room temperature. It is likely that this effect is due to both temperature-dependent alterations in physicochemical properties of the cell membrane and conformational changes in poly-APS resulting in promotion of poly-APS insertion into the membrane and pore formation. However, poly-APS did not deliver siRNA or knockdown protein, whereas both lipofectamine and LipoGen delivered siRNA and produced specific knockdown of functional β-actin similarly. It is not clear why siRNA was not delivered into HEK 293 cells.

## Conclusion

In conclusion, poly-APS was found to be an efficient pore former and at low temperatures (7–12°C) could deliver into cells both cDNA and lucifer yellow but not siRNA. In the future it would be interesting to evaluate the binding of siRNA to poly-APS and its influences on pore formation. It is noteworthy that pyridinium salts are efficient quenching agents reflecting binding properties. Although quenching might have accounted for the negative result obtained when we attempted to deliver fluorescein-labeled siRNA with poly-APS, it does not explain the negative results with siRNA targeted against β-actin. In contrast to poly-APS, LipoGen and lipofectamine did not form Ca^2+ ^permeant pores, but did deliver siRNA into cells and are likely to function in a similar manner. Further studies of the temperature sensitivity to poly-APS might provide approaches, using low temperature and low poly-APS concentrations, to improve efficiency and selectively delivery of macromolecules into cells. In the context of the negative temperature coefficient for poly-APS, it is interesting to note that newly discovered 1,3-dialkylpyridinium and related compounds have been extracted from Arctic species of sponges [19-21]. These findings show that alkylpyridinium compounds are not confined to temperate and tropical Haplosclerida but may contribute to host defence in cold environments.

## Methods

### Materials

Poly-APS (C_13_H_20_N^+ ^monomer M.W. 190.16, [4] with one positive charge per monomer unit) were purified from the Adriatic marine sponge *Reniera sarai *as previously described [4], and dissolved in MilliQ water (stock solution 1 μg/μl) [1-3]. Poly-APS is readily solubilized in distilled water at room temperature and can be kept for months at 4°C without loosing biological activities. We have never observed any precipitation at 4°C when poly-APS was kept at concentrations up to 1.5 mg/ml. However, the solubility is highly dependant on the polarity of the solvent and poly-APS is only poorly soluble in methanol, and even less soluble in ethanol. There is only very limited data on phase behaviour of poly-APS in water. Experiments with dynamic and static light scattering [4] have shown that poly-APS exists as a mixture of monomolecular polymers, creating large supramolecular spherical aggregates with a hydrodynamic radius of 23 nm.

*N*^4^,*N*^9^-dioleoylspermine (LipoGen) [5,7] was synthesized by the procedure described elsewhere [16]. LipoGen was dissolved in ethyl alcohol (stock solution 2 μg/μl). Buffers and NaCl solution were also made up in MilliQ water and buffers were adjusted to 7.4 with aq. NaOH solution.

Lipofectamine was purchased from Invitrogen, U.K. pEGFP (4.7 kbp) and pGL3 (5.3 kbp) DNA were prepared according to the Maxiprep plasmid amplification and purification protocol and stored in a freezer before use. Calf thymus DNA was purchased from Sigma-Aldrich U.K. Foetal calf serum (FCS), 10 × Minimum essential media (MEM), Penicillin G, Streptomycin, L-glutamine, 7.5% NaHCO_3_, trypsin, PBS, and serum-free MEM (Opti-MEM) were obtained from Gibco-Invitrogen.10% Media used during transfection complexes addition was either NaCl-based saline or serum-free MEM. Other chemicals used were supplied from Sigma-Aldrich U.K.

### DNA condensation

The displacement assay fluorescence measurements were run with Perkin-Elmer Fluorescence Spectrophotometer Model LS50B (λ _ex _= 260 nm, λ _em _= 600 nm, 1 cm pathlength, 3 ml glass cuvette, slit width 5 nm) with FLWinLab version 2.00 for data processing. DNA concentration and purity were carried out with a Helios UV spectrophotometer at 260 nm (for DNA concentration) and 280 nm (for protein concentration) prior to the experiments [20]. DNA (6 μg) was obtained from the stock solution and diluted to 3 ml with low-NaCl HEPES buffer (2 mM HEPES, 20 mM NaCl, 10 μM EDTA, pH 7.4) in a glass cuvette stirred with a micro-flea. Immediately prior to analysis, EthBr solution (3 μl, 0.5 μg/μl) was added to the DNA solution, stirred for 1 min to equilibrate the binding process. Aliquots of poly-APS or LipoGen were added to the stirring DNA solution at the desired ammonium/ phosphate (N/P) ratio and the fluorescence measured after 1 min equilibration [20,21]. Optimal N/P ratios for DNA condensation for poly-APS and LipoGen were at 3.50–4.00 (4.1–4.7 μg/ml) and 1.50–2.50 (3.3–5.5 μg /ml). The emission intensity was reported as the percentage of maximum fluorescence (100 %) when DNA was fully intercalated by EthBr without the DNA binding agents and corrected for the background fluorescence of total EthBr in buffer solution.

For the light scattering assay, DNA (60 μg) was taken from the DNA stock solution and diluted to 3 ml with HEPES buffer (2 mM HEPES, 20 mM NaCl, 10 μM EDTA, pH 7.4) in a glass cuvette stirred with a micro-flea. Aliquots of poly-APS or LipoGen were added to the stirring DNA solution at the desired N/P ratio and the UV absorbance at 320 nm was then measured after I min equilibration [21].

### HEK 293 cell cultures

HEK 293 cells (human embryonic kidney cell line) were maintained in culture in Dulbecco's modified Eagle's minimum essential medium, supplemented with 10 % foetal calf serum, 2 mM L-glutamine, 50 μU/ml penicillin, 50 μg/ml streptomycin and 1 % non-essential amino acids. In preparation for experiments and 24 h prior to experimentation, cells were seeded at a density of 0.5 million cells per 35 mm dish.

### HtTA HeLa cell cultures

HtTA-1 HeLa cells, at 1 × 10^6 ^cells, were grown in 150 ml flask with 25 ml of 10% FCS EMEM (minimum essential media with penicillin, streptomycin, glutamine, sodium bicarbonate, and 10% foetal calf serum) [22-24]. Each 500 ml of media contained 50 ml EMEM (10×), 13.5 ml NaHCO_3 _(7.5%), 5 ml L-glutamine (200 mM), 25000 IU penicillin G, 25000 μg streptomycin and 10% foetal calf serum. The culture was incubated at 37°C, 5% CO_2_. The cells were passaged every 3 days. Trypsinized cells were added to the next passage at 1 × 10^6 ^cells/flask.

### Ca^2+ ^imaging

Ca^2+ ^imaging experiments were conducted at room temperature (approximately 21°C) or 12°C. HEK 293 cells were bathed in a NaCl-based extracellular solution containing in mM: NaCl, 130; KCl, 3.0; CaCl_2_, 2.0; MgCl_2_, 0.6; NaHCO_3 _1.0, HEPES 10.0, glucose 5.0. The pH and osmolarity of extracellular solutions were adjusted to 7.4 and 310–320 mOsmol/l with NaOH and sucrose respectively.

Cultured HEK 293 cells were incubated for 1 h in NaCl-based extracellular solution containing 10 μM fura-2AM (Sigma, 1 mM stock in dimethylformamide) and the effects of poly-APS, lipofectamine and LipoGen on intracellular Ca^2+ ^were evaluated using fluorescence ratiometric imaging as previously described [1-3]. For some experiments, the temperature was changed by perfusing cells with saline at 21°C and 12°C. The temperature was monitored using a thermometer probe (Jenway 2000 series) placed in the bath containing the cells. All data are given as mean ± standard error of the mean and statistical significance was determined using the Student's two-tailed unpaired *t *test.

### Macromolecule and probe delivery protocols

#### cDNA delivery into HEK293 cells

HEK 293 cells were washed with NaCl-based saline or serum-free medium, then exposed to 0.5 μg/ml poly-APS for 5 min and then poly-APS and 2.5 μg/ml cDNA encoding for the expression of enhanced green fluorescent protein (pEGFP; Clontech) for 3 h at 12°C.

Additionally, experiments were conducted using lipofectamine as the transfection reagent. Lipofectamine (4 μg) was mixed with 100 μl of serum-free medium and incubated at room temperature for 5 min prior to adding to 100 μl of serum-free medium containing cDNA (1 μg/ml; N/P ratio 4). After mixing this solution containing transfection reagent and cDNA was incubated for 20 min at room temperature and then added to the cells. The cells were incubated for 3 h at 12°C. After incubation with the transfection reagent and cDNA, serum (to give 10 %) was added. For cells incubated with NaCl-based saline, serum-containing medium was added to replace the saline. Cells were maintained in culture for 24 h before green fluorescent protein was examined using either fluorescence microscopy or confocal imaging.

#### cDNA delivery into HtTA cells

DNA complex solution for each well was prepared from solution A and solution B. For solution A, pEGFP (2 μg) was diluted to 100 μl solution with Opti-MEM medium (serum-free media). Solution B was prepared from LipoGen (5.5 and 11.0 μg for N/P ratios of 2.5 and 5.0 respectively), or lipofectamine (2.53 μl for an N/P ratio of 3.0) was diluted to 100 μl with Opti-MEM medium. Each solution was left at 20°C for 30 min. Solutions A and B were then mixed together and vortexed for a brief period, incubated at 20°C for 20 min for DNA complexing. In 6-well or 12-well tissue culture plates, HtTA 2.5 × 10^4 ^cells/ ml were seeded in 4 ml and 2 ml of 10% FCS EMEM respectively. The cells were incubated at 37°C with 5% CO_2 _in an incubator for 24 h, until the cells were 30 – 50% confluent. Prior to the addition transfection complexes, media was removed and replaced with either NaCl-based saline or Opti-MEM. NaCl-based extracellular solution used in HtTA cell experiments was the same as used in HEK 293 cell experiments. For 12°C experiments, cells were placed in a temperature-controlled refrigerator for 10 min before the transfection. Solutions of DNA complexes (200 μl/well) were added, and then cells were incubated in either 37°C with 5% CO_2 _or at 12°C. The 48 h post-transfection samples was analysed by FACS for enhanced green fluorescent protein level.

#### Lucifer yellow delivery

HEK 293 cells were washed with NaCl-based saline, then exposed to 0.5 μg/ml poly-APS for 5 min and then poly-APS and 1 mM lucifer yellow (Sigma) for 1, 2 and 3 h. Pre-incubations in poly-APS and incubations in lucifer yellow were carried at 7°C, 21°C and 37°C. Cells were washed three times before intracellular loading with lucifer yellow was evaluated by fluorescence microscopy immediately after incubation and again after being placed in culture medium and cultured for a further 24 h. Additionally, the abilities of lipofectamine (4 μg/ml) and LipoGen (4 μg/ml) to load HEK 293 cells with lucifer yellow were determined over 3 h incubation periods at 7°C and 21°C.

#### siRNA delivery

HEK 293 cells were washed with serum-free medium, then exposed to 1.0 μg/ml poly-APS for 5 min and then poly-APS and 200 nM siRNA conjugated with fluorescein (QIAGEN) for 4 to 6 h at 37°C. An additional set of experiments were carried out with 1.0 μg/ml poly-APS and 200 nM siRNA conjugated with fluorescein (QIAGEN) for 5 h at 12°C. The fluorescence within the cells was assessed immediately after incubation using confocal imaging. Additionally, experiments were conducted using lipofectamine and LipoGen as transfection reagents. Lipofectamine (4 μg) or LipoGen (4 μg) were mixed with 100 μl of serum-free medium and incubated at room temperature for 5 min prior to adding to 100 μl of serum-free medium containing 50 nM siRNA conjugated with fluorescein. After mixing, this solution containing transfection reagent and siRNA was incubated for 20 min at room temperature and then added to the cells bathed in 1 ml serum-free medium (final volume 1.2 ml). The cells were incubated for 4–6 h at 37°C before imaging siRNA intracellular delivery.

Knockdown of β-actin protein with β-actin specific siRNA (Ambion) was assessed using Western blot analysis. HEK 293 cells were treated and incubated with transfection reagents (poly-APS, lipofectamine or LipoGen) as above with 100 nM siRNA for lipofectamine and LipoGen or with 333 μM siRNA for poly-APS (ratio 1 μg: 30 pmol siRNA for lipofectamine and LipoGen and 1 μg: 667 pmol siRNA for poly-APS) for 3 or 6 h at 37°C. The N/P values for the siRNA β-actin experiments were approximately 1.3 to 8.8 and 1.6 to 10.8 (2.4 to 0.35 μg/ml siRNA to 4 μg/ml lipofectamine or LipoGen respectively). Serum was then added (to give a final concentration of 10 %) and then the cells were maintained in culture for 24 or 48 h. siRNA negative controls were run with non-homologous siRNA. Cells were placed on ice and processed for Western blot protein analysis (Santa Cruz Biotechnology Inc.). Loading controls were conducted by stripping the blot and re-probing using antibodies for NFKB (Cell Signalling).

## Abbreviations

pEGFP plasmid encoding for enhanced green fluorescent protein

EthBr ethidium bromide

LipoGen *N*^4^,*N*^9^-dioleoylspermine

NVGT non-viral gene therapy

Poly-APS polyalkylpyridinium salts

SAINT Synthetic Amphiphile INTeraction

siRNA small interfering RNA

## Competing interests

The author(s) declare that they have no competing interests.
